# An Immersive Virtual Reality Platform for Assessing Spatial Navigation Memory in Predementia Screening: Feasibility and Usability Study

**DOI:** 10.2196/13887

**Published:** 2019-09-03

**Authors:** Kiran Ijaz, Naseem Ahmadpour, Sharon L Naismith, Rafael A Calvo

**Affiliations:** 1 Centre for Health Informatics, Australian Institute of Health Innovation Macquarie University Sydney Australia; 2 School of Electrical and Information Engineering The University of Sydney Sydney Australia; 3 Sydney School of Architecture, Design and Planning The University of Sydney Sydney Australia; 4 School of Psychology The University of Sydney Sydney Australia; 5 Dyson School of Design Engineering Imperial College London London United Kingdom

**Keywords:** virtual reality, healthy aging, memory, cognition, dementia

## Abstract

**Background:**

Traditional methods for assessing memory are expensive and have high administrative costs. Memory assessment is important for establishing cognitive impairment in cases such as detecting dementia in older adults. Virtual reality (VR) technology can assist in establishing better quality outcome in such crucial screening by supporting the well-being of individuals and offering them an engaging, cognitively challenging task that is not stressful. However, unmet user needs can compromise the validity of the outcome. Therefore, screening technology for older adults must address their specific design and usability requirements.

**Objective:**

This study aimed to design and evaluate the feasibility of an immersive VR platform to assess spatial navigation memory in older adults and establish its compatibility by comparing the outcome to a standard screening platform on a personal computer (PC).

**Methods:**

VR-CogAssess is a platform integrating an Oculus Rift head-mounted display and immersive photorealistic imagery. In a pilot study with healthy older adults (N=42; mean age 73.22 years, SD 9.26), a landmark recall test was conducted, and assessment on the VR-CogAssess was compared against a standard PC (SPC) setup.

**Results:**

Results showed that participants in VR were significantly more engaged (*P*=.003), achieved higher landmark recall scores (*P*=.004), made less navigational mistakes (*P*=.04), and reported a higher level of presence (*P*=.002) than those in SPC setup. In addition, participants in VR indicated no significantly higher stress than SPC setup (*P*=.87).

**Conclusions:**

The study findings suggest immersive VR is feasible and compatible with SPC counterpart for spatial navigation memory assessment. The study provides a set of design guidelines for creating similar platforms in the future.

## Introduction

### Background

Dementia, the umbrella term for age-related disorders characterized by a decline in cognitive ability, is expected to double approximately every 20 years to affect 74.7 million people by 2030 and 131.5 million people by 2050 [1]. Efforts to improve diagnosis, treatment, and support are growing ever more important. Dementia is generally preceded by a predementia stage known as mild cognitive impairment (MCI), an intermediary stage on the continuum between age-adjusted healthy cognitive ability and dementia [2]. At this stage, daily activities can still be performed with minimal difficulty, and there is scope for intervention to impede further deterioration [3]. Any method to support the diagnosis of MCI as early as possible could therefore be of great benefit to millions of people. As such, older adults, some of whom seemingly healthy, are often referred for screening.

Neuropsychological tools for screening predementia stages are costly and not always accurate [4]. As one of the earliest clinical manifestations of cognitive impairment is topological disorientation [5], spatial navigation memory tests are used for diagnosis. These tests are generally conducted with pen-and-paper tasks, such as the Mini-Mental State Examination [6]. Computer administrated tests [7] and use of virtual reality (VR) [8] are explored more recently.

VR systems have been used as assessment tools [9,10] for physical activity [11], cognitive assessment [12], and balance assessment [13]. Studies on nonimmersive virtual environments (VEs) found those effective for assessments [8] because of being accessible and feasible while providing controlled settings for conducting cognitive sessions. Recent studies have shown that VR can facilitate information recall [14-16], an important consideration for measuring cognitive decline. It is therefore important to explore immersive VR as an important technology for spatial navigation because of its ability to map real-world functioning [17]. In addition, it can enhance episodic memory, that is, autobiographical memory of past temporal events, in elderly [18] and is deemed feasible as a cognitive training tool [19].

A VR platform can equip clinical neuropsychologists with a feasible assessment technology on which the setting (and therefore the assessment outcome) is generalized to real-life settings [20]. This requires that human-computer interaction (HCI) and VR technology researchers work closely with clinicians to develop new forms of interactions, such as *critical dementia* proposed by Lazar et al as a “lens onto the ways people with dementia are positioned and engaged by the field of HCI” [21]. Such attempt mandates a close study of the needs and requirements of the potential users to create a system with good usability. On the basis of the above, we examine the feasibility of VR technology for mediating information recall test. A number of studies provide evidence to support that choice and guide our research on the VR testing platform for older adults proposed in this paper, as outlined below.

### Mental Models in Virtual Reality as a Mediator of Contextual Representation

Paper-based cognitive assessments, particularly spatial tests, are often a departure from realistic situations [5] and familiar mental models of individuals in everyday life environments. It has been long established that spatial orientation requires identifying many cues such as self-to-surrounding relationships and object-to-object spatial relations [22]. Several studies suggest that VR with realistic settings aids better information recall for both spatial and episodic memory [14,23,24]. Furthermore, head-mounted displays (HMDs) have the potential to induce a sense of presence in the VE, that is, the perception of *being there*.

### Factors Influencing User Engagement

Several factors often impact user engagement with VR including presence, gamified designs [11,25], and natural body interactions [26,27]. Motion sickness, despite recent improvements in VR technology, still has a negative impact on user engagement [12,28,29]. Furthermore, older adults have additional requirements that need to be considered in designing the system: most commercially available HMDs are heavy [30,31], have nonintuitive controllers [32,33], can cause stress or hesitation [33], and large proportion of VR content not specifically designed for this cohort [32,33], all of which negatively influence experiences and engagement of older adult users with the system. There are constant improvements in VR devices and controllers (eg, stand-alone HMDs); however, more investigations are required to design and develop engaging content and interactions suitable for older adult users. Finally, and more relevant to our study, better engagement with the system will likely influence the memory test outcomes in VR and contribute to a better sense of well-being for the individual involved in predementia screening.

### Factors Influencing Perceived Usability and Competency of the Device and Environment Relevant to User’s Capabilities

A number of VR systems are designed for adult users [12,25,26,29,34] without direct consideration of their specific usability needs. Needless to say, such considerations become more significant in systems that aim to assess memory and detect cognitive impairment in older adults. In addition to impeding user engagement, poor usability in a cognitive test could impact the assessment results and consequently disrupt the validity of the outcome. Furthermore, poor usability of system controls can hinder the user’s sense of presence and, subsequently, the experience of the system [35]. Most developers apply general usability principles and techniques such as the classic Nielsen’s heuristic evaluations [36], recommendations on usability for VE [37], and tailoring walkthrough methods for nonimmersive VR applications [38]. Current literature has focused on 3D environments, largely covering nonimmersive VR and younger adults of these systems. However, these should be extended to include older users’ competencies [39] and usability constraints of HMDs, new controllers, and motion trackers for older users. Currently, there is a noticeable lack of such evidence in usability practices for immersive VR.

### Factors Influencing User’s Stress and Motion Sickness

Another area of inquiry is the propensity of VR for causing motion sickness and, subsequently, stress. As cognitive disabilities (eg, dementia) often correlate with other mental health problems such as depression [40], it is important to mitigate the effect of any additional stress. Although designing VR environments for older adults and, in particular, those with memory deficit may highly benefit the screening process, it is important that the cognitive load is manageable, and the negative mental impacts on users are minimized. Researchers have found older adults tend to be reluctant to use new forms of technology [41] that can induce simulator sickness [42] and find immersive VR devices, controllers, and environments intimidating. Providing natural interaction styles with the system, with clear instructional tutorials to familiarize users with the VR device and environment, as well as the VR task, might reduce the stress level. Furthermore, motion sickness can have a detrimental impact on assessment validity or result in discontinuation of the assessments before completion. To reduce motion sickness, users need to have control over their navigation, while avoiding sudden head movements, and exposure to unexpected changes in scenery and orientation.

This paper contributes to the design and evaluation of VR-CogAssess, a new VR platform using photorealistic imagery to assess topological cognitive impairment (ie, spatial navigation memory) as a tool for predementia diagnosis. We test VR-CogAssess with older adults to explore 3 goals. First, we investigate the compatibility of VR-CogAssess compared with a standard personal computer (SPC) setup in an experiment. We assess that based on participant’s performance in landmark recall test using measures such as recall of challenging locations, test duration, and perceived presence in VE. Second, we explore the scope of usability considerations needed for VR memory assessment platforms for older adults to support their interaction. To achieve that, we examine the efficiency of system controls and participant’s perceived enjoyment of using the system. Finally, we study the feasibility of using a VR platform as a memory assessment tool for spatial navigation for older adults. This was assessed based on overall test session time, alignment of the system with computer abilities of the users, and user’s level of stress, which is critical for supporting their well-being. We posit that a VR memory test platform should be designed to accommodate users’ abilities and computer literacy, without distracting them during the test (for instance, because of the novelty of the system) as that might result in users underperforming in their test or abandoning it before completion.

This is a very underinvestigated research area. Our study has the unique advantage of exploring the feasibility of VR using a photorealistic VE for spatial navigation assessment with older adults. Developing such a system is less complicated and more cost-effective than developing a full 3D environment. To achieve that we identify a set of propositions for designing VR systems for older adults that address their needs and then develop VR-CogAssess based on those user needs. An introduction to the propositions and the platform is presented in Methods section, along with details of a study where we examined the feasibility and the usability of the system. On the basis of the results, we discuss those propositions and further considerations for future studies.

## Methods

### The VR-CogAssess Platform

In this section, we describe the VR-CogAssess system and identify a set of 5 design propositions based on the literature and our own design experiences during the project to meet the unmet needs of older adults in technology use. Those propositions guided our design and development process and selection of components. Where applicable, we refer to those as *design propositions* followed by a number.

#### Architecture Overview

**Figure 1:**
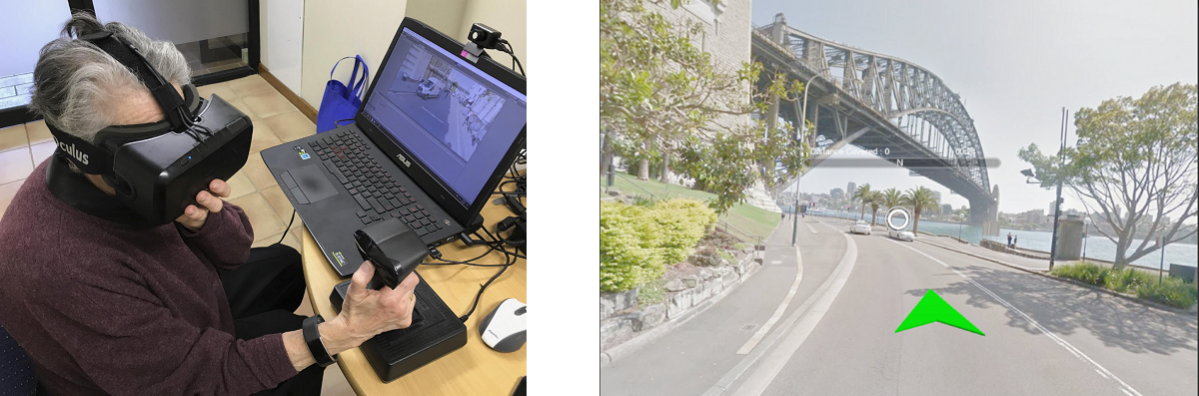
VR-CogAssess Platform (left) and virtual environment interface with 360-degree panoramas(right).

VR-CogAssess is built using the Unity game engine, and its library and controllers are written in Microsoft C#. Three-dimensional visuals are fetched from the Google Street View application programming interface (API) and rendered visually as 360° panoramas to the user through an Oculus Rift VR HMD. A Microsoft Band smartwatch reads physiological signals including heart rate variability (HRV) and galvanic skin response (GSR) during the assessment task. These data are then sent to an Amazon Web Services cloud service for storage through an Android mobile phone app to supplement analysis on stress. Finally, the platform supports user navigation of the VR environment through a CH Products Flightstick joystick. The setup of VR-CogAssess with main components and visual interface are shown in (Figure 1).

The VE interface is rendered through the HMD and provides a photorealistic environment with a continuous field of view around the user. Unlike SPC displays where users typically control the directional view by manually pressing left or right on a keyboard, the VR system enables the users to simply turn their head in the desired direction as the HMD monitors axis rotation against a reference point. Taking elementary usability considerations into account such as larger font and object sizes, we also designed the interface by identifying and incorporating guidelines for older users. Over multiple iterations, we then revised the system based on input and feedback from clinical neuropsychologists with extensive experience in cognitive assessment for individuals living with MCI. The simplicity of the interface is by design, for which we used a set of design propositions, as shown in Textbox 1. We then describe how those propositions were integrated into the design of VR-CogAssess. Of note, we do not aim to validate these design propositions generated from the literature; rather, we examine their feasibility to design a VR assessment system for older adults.

**Design propositions for immersive virtual reality–based memory assessment systems.**

- Design proposition 1: designing for simplicity should consider reducing interface features to the minimum necessary for the task [43].
- Design proposition 2: physical gestures to operate navigation controller should be paralleled with those of the virtual space to cater for optimum hand-eye coordination. It should also allow sensitivity calibration to control the speed of user navigation in virtual environment.
- Design proposition 3: to achieve a sense of control and avoid stressful interaction, the system should be optimized to provide users with a sense of autonomy and choice [44].
- Design proposition 4: the system should teach the user 1 skill at a time and have multiple controlled, momentary stops during the interaction so that users can seek support from the test facilitator [45].
- Design proposition 5: the platform should be customizable to accommodate new memory test protocols with personalized features.

#### Visual Interface

The design proposition 1 (Textbox 1) was implemented in the interface by limiting the task status cues. These include distance covered, time spent, a compass bar for assisting orientation, and a white indicator to point users to the locations they can go to. To simplify, this interface only highlights the interaction that is task related. To further improve the usability, the interface also repeatedly displays textual captions such as instructional hints or reminders about remaining time.

#### Navigation Controls

The navigation control in the VR-CogAssess environment is achieved using a joystick. Tilting the joystick allows the user to change the angle of view for turning corners, and pressing a large button allows forward movements in VE.

Vallejo et al [46] compared 3 different button controllers including the Razer Hydra motion sensing controller and touchpad and found that a joystick was preferred by the participants in navigation tasks. Given the reduced fine motor control and hand-eye coordination skills in older adults [43], our platform allows sensitivity calibration to control the speed of navigation in VE, based on design proposition 2 (eg, movement speed and transition to next locations).

Given that older users at risk of dementia might lack skills for operating computers or other interactive systems, it is essential to provide them with a sense of agency and volition for system’s controls to support these interactions. This will contribute to their enjoyment and engagement with the system [47,48] and could potentially reduce performance anxiety. In summary, our system supports the sense of agency in navigating the environment based on design proposition 3 in a way that reduces confusion and allows a feeling of being in control of the navigation. Users are free to move in any direction with a natural tilt angle of the controller and a press of a button.

#### Task and Tutorial Implementation

The task involves a landmark recall test designed by author SN at the Brain and Mind Centre, The University of Sydney. Users navigate the platform environment from a starting location and are asked to identify 6 landmarks. The landmarks are scattered at different points along a designated navigation path and intentionally vary in difficulty (including 2 challenging landmarks) to locate to support performance discrimination. To avoid confounding spatial navigation ability with the user’s ability to use the technology, we implemented an introductory tutorial (5-min long). The tutorial provides a computer-synthesized voiceover for delivering the instructions for completing the task. This reduces the workload of the facilitator and enforces a level of consistency in the information received across participants.

The system displays on-screen textual hints and audio instructions, one at a time aligned with design proposition 4. There is considerable scope for task customization in this platform guided by design proposition 5, as any location available in the far-reaching Google Street View API can be used [49]. The Unity controllers for managing the environment to the HMD is also written to allow other APIs. Owing to this modifiability, the platform can be repurposed to other potential spatial navigation assessment tasks. For example, although the current task is designed to assess spatial navigation ability in an unfamiliar location to the user, the location can be personalized. This can be an immediate vicinity of the user’s home, neighborhood, or city, which may assist health care practitioners in evaluating or providing intervention based on how a person performs in a familiar setting.

### Study

In this section, we describe the 2 conditions of the study: (1) we use VR-CogAssess for a landmark recall memory test and (2) we use an SPC setup. The VR-CogAssess platform was designed with clinical neuropsychologists to capture task variables including task duration, date, distance traveled, navigational mistake count, landmark recall count, and speed and auxiliary data such as user details, heart rate, and GSR. VR-CogAssess facilitates data collection for clinical practitioners by capturing multitudes of variables mentioned above. Data collection and processing are managed automatically in VR-CogAssess to reduce workload, cost, and save time. For example, counting navigational mistakes would require the test administrator to rigorously monitor participants for the whole duration of a task. Instead, a Unity controller is programmed to record the identification of locations visited outside a prerecorded correct path. Furthermore, the actual trail taken by the participant is visualized on task completion based on recorded sequence taken. Physiological data on HRV and GSR from a Microsoft Band is timestamped and recorded in XML format and stored on a cloud server. This captures changes in the stress level of participants, as excessive stress can be detrimental to confidence on assessment scores.

In SPC condition, users were expected to navigate the same locations in recall test as was assigned to VR users. However, there were differences in navigation controls (standard keyboard arrow keys were used in SPC), and the visual interface was a standard monitor. A 5-min long tutorial was presented to SPC users, same as in VR condition, to familiarize them with navigation controls and test environment. In summary, the 2 conditions presented the same locations in recall test, but there were differences in navigational controls, data recording method (administered by researcher), and the visual interface.

We investigated the following research questions (RQs) aligned with our initial aims of evaluating immersive VR platform for spatial navigation.

- RQ1: Are there any differences in assessment outcomes between the VR and SPC conditions?
- RQ2: Are there any usability differences between the VR and SPC conditions?
- RQ3: Is it feasible to use VR-CogAssess for older adults to complete the memory test assessment with minimal stress level, given their computer skills and competency?

### Participants

Participants were recruited at a community center in Sydney, Australia. Participants were healthy older adults (N=42; mean age 73.22 years, SD 9.26). They all gave informed consent for this study approved by the Human Ethics Committee at authors’ university (protocol #2016/629). Participants were randomly allocated to 1 of the 2 study conditions, whereby they completed a landmark recall test using either the VR-CogAssess platform with the joystick control (VR, n=22) or a standard computer setup running Google Street View (SPC, n=20). Both conditions shared a similar starting point, environment location, and the recall assessment task.

Table 1 summarizes the demographic information on age and education distribution across the conditions. There were no significant differences in age (*P*=.30) and education level (*P*=.11) between participants in the 2 conditions.

None of the participants had been diagnosed with MCI, Alzheimer disease, and other dementias. Similarly, no participant had recently visited Cambridge or had lived there for more than a month.

**Table 1:** Participants’ demographics in 2 study conditions.

Demographics		Standard personal computer (n=20), n (%)	Virtual reality (n=22), n (%)
**Age (years)**			
	50-59	2 (10)	2 (9)
	60-69	4 (20)	8 (36)
	70-79	7 (35)	8 (36)
	80-89	7 (35)	4 (18)
**Education**			
	Postgraduate	3 (15)	7 (31)
	Undergraduate	6 (30)	9 (40)
	Technical college	4 (20)	5 (22)
	High school	6 (30)	1 (4)
	Primary school	1 (5)	—^a^

^a^Missing data.

### Data Collection

All participants followed 7 steps: (1) study introduction, (2) pretest questionnaire, (3) platform learning, (4) landmark memorization, (5) minitest, (6) landmark recall test, and (7) posttest feedback. The estimated study session was kept between 30 and 45 min.

#### Study Introduction

At the start of the session, participants were introduced to the study and gave informed consent. Participants then proceeded with the next step to complete the pretest questionnaire.

#### Pretest Questionnaire

In a pretest questionnaire, participants were asked to provide information about age, highest level of education, and basic computer skills proficiency using a range of options (see Table 1). Participants then proceeded with learning about the platform.

#### Platform Learning

**Figure 2:**
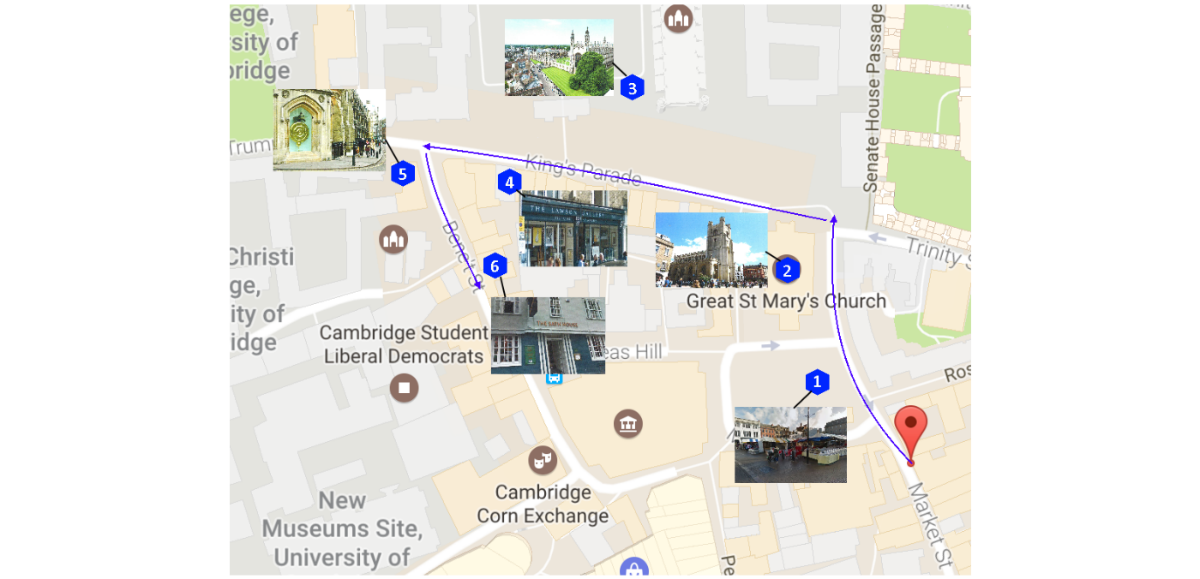
Two-dimensional map of Cambridge shown with 6 landmarks, navigation route, and starting point.

This step provided participants with essential skills to complete the upcoming landmark recall test and become familiar with the relevant platform. Participants completed 1 of the 2 tutorials depending on the study condition they were randomly assigned to. The tutorials shared the same location (Sydney Harbour Bridge; Figure 2) and involved learning the same basic navigation actions (move forward or back and turn left or right). In SPC condition, the standard keyboard up, left, and right arrow keys were used as navigation controls, whereas in VR condition, a joystick was used. Moving forward in VR was achieved by pressing a front trigger with the thumb and turning involved tilting the joystick left or right. Participants learned these navigation controls over a series of instructions that involved moving toward a bridge, turning to identify another landmark, and then moving back to the original location. This step was verbally administered by the researcher in the SPC condition, whereas VR condition had built-in tutorial with synthesized voice for guiding participants. All participants were asked to wear a Microsoft Band 2 to track physiological signals before and during recall part of the test. This allowed the researchers to record baseline signals before the test phase.

#### Landmark and Navigation Memorization

This step required participants to memorize the appearance of and navigation path to 6 landmarks. All landmarks were in Cambridge, United Kingdom, and were selected because they would be interesting but in a foreign country (therefore less likely that participants would know the place well). These landmarks (highlighted in Figure 2) were of different levels of difficulty: easy (Street Market & King’s College), moderate (Great St. Mary’s Church, The Corpus Clock), and 2 challenging locations (Lawson Gallery and Bath House), which required multiple turns or were not easy to find. This provided the foundation for the main recall test. Materials used for this step included a 2D printed and laminated map and 6 individual landmarks pictures; those were identical in both conditions with the same test location and starting point for consistency (Figure 2).

We first explained the 2D map: starting location, path, and the 6 landmarks. The landmarks were located on different points along a path and were intentionally chosen to create various difficulty levels. Large images of the 6 landmarks were also printed and made available to the participants. We informed the participants that landmark name and number were not required for information recall test. Participants could go back at any time if they missed a landmark during the task. Although a map was available for the memorization phase, individual landmark pictures were shown to participants one by one for only 10 seconds at a time. On presentation of each landmark, its location was also indicated on the map. This was to ensure that the participant relied on their memory the landmark location (rather than its appearance) in the main recall phase. The process was repeated twice to provide participants with sufficient opportunity to memorize the location and appearance of landmarks.

**Figure 3:**
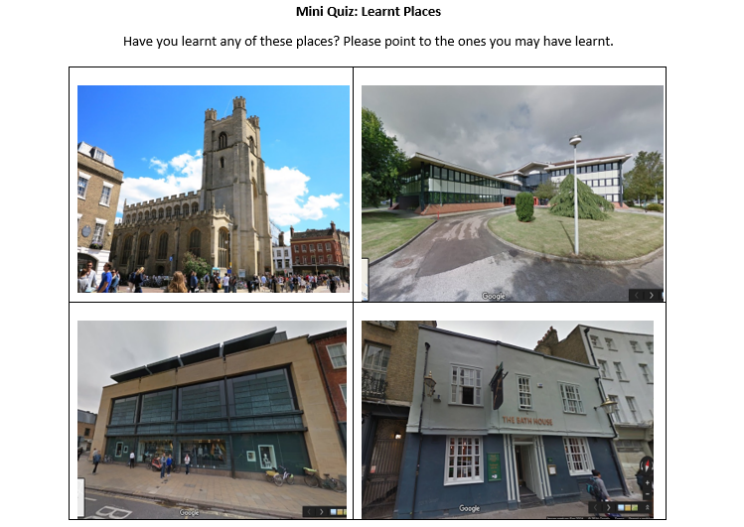
A version of mini quiz with combination of correct and incorrect landmarks.

#### Mini Quiz

Participants were then asked to complete a paper-based mini quiz to identify the landmarks learnt in the previous step. Participants who could not identify at least one landmark correctly would be eliminated. This quiz included a combination of 2 correct and 2 incorrect landmarks. As this step involved further opportunities for viewing some of the correct landmarks, different copies of quiz (Figure 3) were used with various permutations of correct and incorrect landmarks. Participants were not informed of their performance on the quiz to not pronounce their landmarks learning at this stage.

#### Landmark Recall Test

Participants were allocated to a condition matching the learning phase and were tasked with identifying 6 landmarks. A brief reminder instructed participants to identify the 6 learnt landmarks within 15 min. Participants could ask any question they might have before the test begins. Physiological signals were recorded for the entire session via an Android App. We also recorded the number of correct landmark recalls, the specific landmarks identified, and number of time navigational mistakes were made (wrong turns made).

#### Posttest Questionnaire

At the end of each session, participants completed a questionnaire to provide an assessment of their perceived competence, presence, intuitive controls, enjoyment or interest, and pressure or tension during the test. These scales are based on a standard questionnaire, Players Experience of Need Satisfaction (PENS) [50] and Intrinsic Motivation Inventory (IMI) [51]. We added an additional open-ended question, “Do you have any other comments, feedback and recommendations?”

## Results

### Assessment Ability and Outcome

The 2 conditions were compared to establish the extent to which navigation performance and assessment scores are comparable in the 2 conditions. This pertains to RQ1 and is based on recorded landmark recall test information, physiological data, and posttest questionnaire.

#### Correct Landmark Recall and Navigation Mistake Count

The number of landmark recalls and navigation mistakes were recorded for participants who were randomly assigned to 1 of the 2 conditions (SPC or VR). We define higher spatial navigation ability based on higher landmark scores and lower mistake counts. Although participants in both conditions had similar cognitive status (did not declare any cognitive impairment before the test), on average, those in the VR condition identified more landmarks correctly and made less navigational mistakes. A *t* test suggests there is a significant difference between the 2 conditions for correct landmark recalls (*t*_40_=−3.02; *P*=.004) and navigational mistakes (*t*_40_=2.11; *P*=.04). Cohen *d* suggests a notably large effect size for landmarks recall count (*d*=0.94) and medium effect for navigational mistakes (*d*=0.65). Table 2 summarizes detailed results.

**Table 2:** Differences in assessment ability, outcome, and usability in 2 conditions (*t* test).

Variables	Standard personal computer, mean (SD)	Virtual reality, mean (SD)	*t* test (*df*)	*P* value
Correct landmark recall	3.4 (1.19)	4.55(1.26)	−3.02 (40)	.004^a^
Navigation mistakes	5.90 (2.36)	4.09 (3.12)	2.11 (40)	.04^b^
Challenging landmark recall 1	0.30 (0.47)	0.50 (0.51)	−1.32 (40)	.20
Challenging landmark recall 2	0.10 (0.31)	0.50 (0.51)	−3.03 (40)	.004^a^
Task duration (min)	10.70 (3.76)	10.45 (3.73)	0.212 (40)	.83
Presence	2.80 (1.88)	4.59 (1.71)	−3.23 (40)	.002^a^
Intuitive controls	4.95 (1.61)	4.45 (1.59)	1.0 (40)	.32
Enjoyment	4.25 (1.65)	5.64 (1.22)	−3.12 (40)	.003^a^
Session time	35.25 (7.0)	33.64 (8.5)	0.67 (40)	.51
Stress	2.80 (1.51)	2.73 (1.39)	0.16 (40)	.87
Heart rate variability (ms)	807 (92)	792 (80)	0.58 (40)	.57
Competence	3.95 (1.64)	4.27 (1.78)	−0.61 (40)	.55

^a^Statistically significant at *P*<.01.

^b^Statistically significant at *P*<.05.

#### Identifying Challenging Landmarks

It was hypothesized that challenging locations might have different recalls in different conditions. Challenging locations were intentionally outside the immediate field of view of the participants with Bath House being particularly challenging, as it required the participant to navigate through multiple streets. We found significant differences (Table 2) for 1 of the 2 challenging landmarks, the Bath House; (*t*_40_=−3.03; *P*=.004) with very large effect size (*d*=0.95). VR-CogAssess closeness to real-world spatial information was also approved as a challenging landmark that involved multiple turns was identified more times in VR condition than SPC.

#### Task Duration

We recorded the task duration in each condition as a measure for the platform-specific assessment ability. We expected participants in VR condition to complete the recall task faster, as the CogAssess platform enables better spatial navigation. However, results (in Table 2) suggest no significant differences between the 2 conditions. This measure, however, may not be very accurate because of certain design decisions made in relation to VR-CogAssess. For example, we intentionally designed a slow joystick turning speed that adds to task duration for maneuvering. Therefore, the number of correct landmark recall and navigational mistakes remain the major measure for assessment ability.

#### Perceived Presence

VR participants (mean 2.80, SD 1.88) reported significantly higher perceived presence (*t*_40_=−3.23; *P*=.002) compared with SPC participants (mean 4.59, SD 1.71), with a very large effect (*d*=0.99). A cross-tabulation further reveals a trend in perceived presence for each condition, where 12 participants in SPC rated very low presence (1-2), whereas 11 participants in VR condition rated very high presence (6-7).

### Usability

In this section, we examine the result from the posttest questionnaire in relation to RQ2: “To what degree is the VR condition usable and enjoyable to the participant?”

In the pretest questionnaire, when asked about technology competencies in SPC condition, 65% (13/20) participants reported beginner level and a need for assistance with Web browsing, emails, and use of keyboard and mouse, whereas 20% (4/20) participants in SPC condition self-reported competent in basic computer skills. In the VR condition, 36% (8/22) participants required assistance, and 59% (13/22) self-reported being competent in computer skills.

#### Intuitive Control

The measures of intuitive control from PENS questionnaire indicate a perception of usability: (1) “learning the task controls was easy,” (2) “the task controls are intuitive,” (3) “when I wanted to do something in the task, it was easy to remember the corresponding control.” On the basis of the results for those questions, we found no significant differences between the 2 conditions. Usability of controls was critical for users to perform landmark recall test quickly and with minimal frustration following a basic pretest tutorial.

After the memory recall test, the average rating reported for intuitiveness of the controls used was lower in the VR condition (mean 4.45, SD 1.59). A cross-tabulation suggests most participants, regardless of the condition they were assigned to, reported midrange scores (SPC=10 and VR=12). Nonetheless, relatively higher number of participants reported high perceived intuitive controls (SPC=8 and VR=7) than low perceived intuitive controls (SPC=2 and VR=3). This suggests almost equal number of participants struggled with controls in both conditions. Only 9% (2/22) participants reported slight motion sickness toward the end of the session after approximately 10 min in VR condition.

#### Enjoyment

Results relevant to enjoyment are summarized in Table 2. On average, aggregate ratings of enjoyment were higher in VR (mean 5.64, SD 1.22) compared with SPC (mean 4.25, SD 1.65), and the difference was significant (*t*_40_=−3.12, *P*=.003, and *d*=0.95).

#### Observations

Observations during the study session and poststudy feedback gave us further insights into participants’ interactions with 2 conditions. Some participants in both SPC and VR condition appeared to struggle initially with controls before reaching to a comfortable level. One participant in VR condition reported slow joystick movements, whereas another said it was not user friendly. The slow joystick reaction was our design decision to mitigate possible motion sickness as a consequence of sudden movements in immersive VR. Slow controller movements aimed to provide control and agency to the older adults. Participants with advanced computer skills noted the slow joystick turns hindered their experience. A walking pad or treadmill was suggested as a replacement to joystick by 1 VR participant. This feedback suggests the value of personalized settings of controllers for users with different competency levels. Being flexible platform, VR-CogAssess is easily modifiable to provide natural interactions; however, feasibility of other controllers needs appropriate evaluation for this cohort. Users’ observed controller interactions in both conditions emphasize the need to extend the initial tutorial for longer period. The tutorial should however provide 1 challenge or teach 1 skill at a time.

### Feasibility and Perceived Competence

A VR platform is feasible (RQ3) when it allows users to complete the assessment session in a reasonable time window, with less stress and matching to their perceived and actual computer competencies. In the VR condition, the recall test took on average 10.45 min (SD 3.73) where the entire session lasted on average 33.64 min (SD 8.5). Stress level during the assessment session was another concern and defining criterion for the feasibility of VR platform. Participants reported the perceived stress they experienced during the assessment task, using the IMI measures in the posttest questionnaire. A *t* test (Table 2) on perceived stress found no significant differences between SPC (mean 2.80, SD 1.51) and VR (mean 2.73, SD 1.39). Notably, participants in both conditions reported mild aggregated ratings of stress (mean<2.80). In addition, we used a wristband monitoring device to record the mean HRV data during the recall test. Similar to perceived stress, we found no significant differences in mean HRV (*t*_40_=0.58; *P*=.57) between the 2 conditions. However, GSR was excluded from analysis because of the presence of noise in the collected data.

Another important factor for feasibility of using the VR condition was the level of computer skills required for cognitive assessment relevant to perceived competencies of the user. Participants self-reported their perceived competence using 3 rating subscales on PENS questionnaire (recently validated for gaming environments [52]). We averaged those ratings and performed a *t* test to examine the differences. There was no significant difference between ratings received from participants in SPC (mean 3.95, SD 1.64) and VR (mean 4.27, SD 1.78). This measure was important to investigate older adults’ capabilities to complete the designed assessment task. The competence questions capture the participant’s perception of their own ability to perform the test on navigating to 6 landmarks. Large range of scores (SPC: SD 1.64; VR: SD 1.78) were observed in competence rating, a cross-tabulation assists to further investigate ratings on individual scale. Participants ratings were separated for low (1-2), mid (3-5), and high (6-7) ranges. However, more VR participants (n=9) self-reported high competence than SPC condition (n=4), where low competence was reported by equal number of participants in both conditions (n=6).

Furthermore, we examined the difference between the landmark recall performance in participants based on their actual computer skills (self-reported in pretest questionnaire). Those who reported computer proficiency as *never used*, *beginner*, and *competent* were compared. We found significant differences (*t*_18_=−2.60, *P*=.02) in landmark count during recall phase between SPC (mean 3.25, SD 1.29) and VR (mean 4.63, SD 0.92) participants when differentiated as beginner based on computer skills. However, these results represent a small sample, as groups were further divided into subcategories, and there could be subjective differences in computer proficiency reported between participants.

## Discussion

### Principal Findings

Our results show that VR-CogAssess is a feasible platform for spatial memory assessment. The results extend previous research on feasibility studies for less-immersive VR setups [17-19] in cognitive assessment and introduce a fully immersive platform to conduct memory recall tests using VR HMD. Our findings reveal that using VR-CogAssess with HMD, when compared with an SPC setup using the same locations on Google Street View, results in better assessment outcomes, probably because of better alignment of the VE with users’ mental models. We base this on the better results we received from participants in the VR group with respect to their correct landmark recall, less navigation mistakes, successful identification of challenging landmarks, and better perceived presence. Furthermore, VR-CogAssess achieves better assessment feasibility compared with the SPC setup, as users in the VR group perceived slightly less stress (although not significantly different to SPC), and their performance competency was comparable with SPC group. Stress-free interactions with novel VR technology are imperative for self-efficacy of older adults, raising their confidence in the task performance [33] and assessment’s acceptability. Experiencing enjoyment and lower levels of stress is highly important in this type of situation, as it may increase the likelihood of people participating in memory screening, particularly in clinical settings, which are often perceived as stressful.

Designing immersive VR experiences for older adults involves several challenges such as limited physical, cognitive, and technical competencies. Design of immersive VR experiences for older adults in general and those who are at risk of cognitive decline specifically requires several iterations. We were able to demonstrate a comparable level of perceived usability between the VR and SPC conditions, despite the relative novelty of VR technology for our participants. This can be attributed to a set of 5 evidence-based design propositions that guided the design of VR-CogAssess platform, such as the need for easy-to-use controls and natural interactions. Initial training plays an important role in giving the users an opportunity to familiarize themselves with VR setup, learn the controls, and understand the basic actions. These interactions should recognize user’s agency during the experience and provide them with choices. User should be given effective and easy tools for exploring the VE aligned with the main goal of spatial navigation. Fulfilling this basic psychological need of users will contribute toward their enjoyment, satisfaction [48], and ultimately to their sense of well-being. Future studies on VR environments for cognitive assessment will benefit from considering the 5 propositions suggested in this paper and from working in interdisciplinary groups.

Diagnostic assessments are generally done in the hospital environment, where individuals undergo a series of time-consuming tests, and the health professionals experience time pressure. Therefore, memory assessment such as landmark recall test needs to be completed in a reasonable time frame without imposing additional distractions for users when they engage with an immersive (and potentially novel) VR environments. For instance, when the VR environment for memory assessment presents virtual objects and stimuli (eg, trees, people, and cars), design should aim for simple interactions with minimal distractions to keep users focused on the main memory recall task. This may pose a dilemma for the designer, as we believe the very same stimuli in VR environments (eg, environmental objects and textures) provide better alignment to the users’ mental models of the real world and therefore increase the validity of the assessment outcome. VR cognitive assessments have the advantage of higher levels of presence and enjoyment (as demonstrated in our results) to engage users in even long assessment sessions, where stress levels needs to be closely monitored. Any attempt to improve presence and enjoyment in VR experiences can therefore assist to keep stress levels in check.

One of the limitations of this study is that feasibility and usability of our platform was tested with an Australian cohort of healthy older adults with no memory issues reported. Although this is the population at risk of dementia that is often referred for screening, it is important to conduct further research in clinical setting and include participants who are readily identified as exhibiting symptoms of memory impairment. We note however that some of our design considerations such as the joystick controller’s speed and interaction with VR environment were made slow to match common competencies of the aged participants; these may have to be adjusted for visitors in clinics, particularly those with musculoskeletal complaints or neurodegenerative diseases that limit movement or are characterized by tremor such as Parkinson disease. Simultaneously, it is possible that users with better computer skills may find the design not matching their competency level and experience disinterest.

In the future, we intend to further investigate VR-CogAssess in clinical setting and include a comparison of diagnostic accuracy and usability between healthy older adults and individuals with MCI. Another worthwhile endeavor would be to conduct a longitudinal study to study whether VR-CogAssess can monitor a participant’s memory decline or recovery; this may require configuring the task to use a familiar location and monitoring performance changes over time. A strength of using Google Street View is that the platform can easily be modified to use nearly any location. This ability to contextualize the test allows user personalization and might be valuable to study.

### Conclusions

Dementia is a complicated disease that can be detected using novel assessment tools and technologies developed through multidisciplinary efforts of HCI researchers and clinical neuropsychologists. In this paper, we introduced VR-CogAssess, a new platform for assessing spatial navigation memory in older adults. The evaluation compared the VR platform with SPC, a desktop setup, and involved healthy older adults. The VR participants achieved higher landmark recall scores, reported higher levels of presence, and enjoyed the task more when compared with SPC participants. The VR participants also perceived slightly less stress, suggesting better accommodation of mental health needs of older adults when memory assessment is administered through VR technology. These findings are promising, showing the feasibility of our immersive VR platform as a potential tool for cognitive assessment based on spatial navigation memory.

This study focused on the design and evaluation of VR-CogAssess, proposing a set of 5 design propositions for maintaining a reasonable level of usability for older adults compared with SPC setups. These propositions encourage VR systems’ design that consider aging population needs and contribute to their well-being. In a future iteration of VR-CogAssess, we plan to allow customized controller speed based on individual needs and skills.

## Abbreviations

API: application programming interface
GSR: galvanic skin response
HCI: human-computer interaction
HMD: head-mounted display
HRV: heart rate variability
MCI: mild cognitive impairment
PENS: Players Experience of Need Satisfaction
RQ: research question
SPC: standard personal computer
VE: virtual environment
VR: virtual reality
